# Diagnoses and mortality among prehospital emergency patients calling 112 with unclear problems: a population-based cohort study from Denmark

**DOI:** 10.1186/s13049-022-01052-y

**Published:** 2022-12-12

**Authors:** Stine Ibsen, Karoline Bjerg Dam-Huus, Christian H. Nickel, Erika Frischknecht Christensen, Morten Breinholt Søvsø

**Affiliations:** 1 CPER - Centre of Prehospital and Emergency Research, Aalborg University Hospital and Institute for Clinical Medicine, Aalborg, Denmark; 2 Department of Physiotherapy, University College of Northern Denmark, Aalborg, Denmark; 3 Department of Emergency and Trauma Care, Centre for Internal Medicine and Emergency Care, Aalborg University Hospital, Aalborg, Denmark; 4 Prehospital Emergency Services, North Denmark Region, Aalborg, Denmark; 5 Emergency Department, University Hospital Basel, University of Basel, Basel, Switzerland

**Keywords:** Prehospital emergency care, Non-specific complaints, Diagnoses, Mortality, Unclear problem, Symptoms, Emergency care

## Abstract

**Background:**

Patients calling for an emergency ambulance and assessed as presenting with ‘unclear problem’ account for a considerable part of all emergency calls. Previous studies have demonstrated that these patients are at increased risk for unfavourable outcomes. A deeper insight into the underlying diagnoses and outcomes is essential to improve prehospital treatment. We aimed to investigate which of these diagnoses contributed most to the total burden of diseases in terms of numbers of deaths together with 1- and 30-day mortality.

**Methods:**

A historic regional population-based observational cohort study from the years 2016 to 2018. Diagnoses were classified according to the World Health Organisation ICD-10 System (International Statistical Classification of Diseases and Related Health Problems, 10th edition). The ICD-10 chapters, R (‘symptoms, signs and abnormal clinical and laboratory findings, not elsewhere classified)’ and Z (*‘*factors influencing health status and contact with health services”) were combined and designated “non-specific diagnoses”.

Poisson regression with robust variance estimation was used to estimate proportions of mortality in percentages with 95% confidence intervals, crude and adjusted for age, sex and comorbidities.

**Results:**

Diagnoses were widespread among the ICD-10 chapters, and the most were ‘non-specific diagnoses’ (40.4%), ‘circulatory diseases’ (9.6%), ‘injuries and poisonings’ (9.4%) and ‘respiratory diseases’ (6.9%). The diagnoses contributing most to the total burden of deaths (n = 554) within 30 days were ‘circulatory diseases’ (n = 148, 26%) followed by ‘non-specific diagnoses’ (n = 88, 16%) ‘respiratory diseases’ (n = 85, 15%), ‘infections’ (n = 54, 10%) and ‘digestive disease’ (n = 39, 7%). Overall mortality was 2.3% (1-day) and 7.1% (30-days). The risk of mortality was highly associated with age.

**Conclusion:**

This study found that almost half of the patients brought to the hospital after calling 112 with an ‘unclear problem’ were discharged with a ‘non-specific diagnosis’ which might seem trivial but should be explored more as these contributed the second-highest to the total number of deaths after 30 days only exceeded by ‘circulatory diseases’.

**Supplementary Information:**

The online version contains supplementary material available at 10.1186/s13049-022-01052-y.

## Background

To support early symptom-based care in emergency medicine, management protocols for procedures and treatments are often available for specific complaints. However, acutely ill patients do not always present with specific symptoms such as chest pain, instead, they present with non-specific complaints such as generalised weakness or tiredness [1]. Patients with non-specific complaints are well known in the Emergency Department (ED) setting and are at increased risk of receiving an incorrect diagnosis [2] and for non-favourable outcomes, such as the increased risk of hospital admission [3, 4], morbidity [5] and 30-day mortality [1, 4, 6]. Studies investigating non-specific complaints demonstrate that most patients with non-specific complaints are older [1]. Older patients are the fastest-growing population in emergency care [7, 8] and consequently, non-specific complaints are of increasing importance.

However, the EDs are not the first to see all patients as the most urgent patients call the emergency number to get help from the emergency medical services (EMS). In the prehospital field, the approach to assessing and treating the patients is based on the chief complaint. Thus, unclear complaints can be difficult to handle for EMS, especially as research points towards a high risk of serious outcomes among this group. EMS play an important role in assessing, initiating treatment, and transporting patients to the ED. In Denmark, when patients call for an ambulance the Danish Index for Emergency Care (Danish Index) is used to support the health care professionals’ (call handler) assessment of the patients’ complaints or mechanism of injury, the severity and level of urgency. The Danish Index encompasses the criterion ‘unclear problem’ for patients calling with symptoms that cannot be categorised as a specific criterion such as chest pain or breathing difficulty [9]. Previous studies have reported ‘unclear problem’ to account for a considerable part of all emergency calls, ranging between 11 and 19% [6, 10, 11]. We recently documented that ‘unclear problem’ was the third most deadly criterion for patients calling for an ambulance, only exceeded by ‘possible cardiac arrest’ and ‘breathing difficulties’ [6].

A deeper insight into the underlying causes behind, such as diagnoses and the impact of demographics as well as comorbidity is necessary before we can develop interventions to improve prehospital triage and treatment of this patient group. Therefore, we performed an observational study of hospital diagnoses for patients calling for an ambulance who was assigned the criterion ‘unclear problem’. We aimed to investigate which of these diagnoses contributed most to the total burden of diseases in terms of numbers of deaths together with 1- and 30-day mortality.

## Methods

### Study design

We conducted a retrospective historic population-based observational cohort study on routinely collected healthcare data from the years 2016 to 2018. We followed The Strengthening the Reporting of Observational Studies in Epidemiology (STROBE) Statement [12].

### Setting

In Denmark, emergency 112-calls that relate to medical emergencies are forwarded to an Emergency Medical Coordination Centre. The emergency calls are answered by health care professionals who are registered nurses or paramedics, peer-trained to handle the emergency call. The healthcare professionals assess the severity of the condition according to the patient’s symptoms or mechanism of injury and determine the response supported by the Danish Index. The Danish Index is a decision-support tool to categorise each emergency call into the 37 main criteria describing specific symptoms e.g. ‘breathing difficulties’, or mechanisms such as traffic accidents, and one criterion for those presenting with ‘unclear problem’ [10]. Each criterion is subdivided into five urgency levels (A-E) for the ambulance response. Level A describes life-threatening conditions or potentially life-threatening conditions, level B is urgent but not life-threatening conditions, level C is non-urgent conditions that require an ambulance, level D is non-urgent conditions requiring supine patient transport, and level E are conditions, requiring medical advice only [9]. The healthcare professionals have the option to advise the patient over the phone, refer the patient to a primary care provider, or dispatch an ambulance. As an ambulance arrives at the scene the ambulance personnel examine the patient and decide whether to bring the patient to the hospital for further evaluation or to treat and leave the patient on the scene, depending on the patient’s condition. The decisions to treat and leave the patient are made in consultation with a prehospital physician. When a patient is admitted to a hospital in Denmark, it is required that that patient receives a diagnosis within Danish SKS Classification which corresponds to the International Classification of Diseases and Related Health Problems, 10th edition (ICD-10) [13].

This study included data from the North Denmark Region with both urban and rural areas and approximately 590,000 inhabitants, corresponding to 10% of the Danish population[14]. The Danish healthcare system including EMS is free for all citizens [13]. The North Denmark Region has three emergency departments of which one is a trauma centre.

### Participants

We included patients who were assigned the criterion ‘unclear problem’ at the time of an emergency call and brought to the hospital by ambulance. Patients without hospital contact were excluded from the study as they do not receive a diagnosis in the hospital. Each Danish citizen has a unique 10-digit civil registration number used in all Danish registries, which was used to link data between registries. We excluded patients without residence in Denmark, patients from other regions and patients without registered civil registrations number.

### Variables and outcome

Demographic data on the study population, age, gender and comorbidities, were obtained and diagnoses were classified according to the World Health Organisation ICD-10 System. Comorbidities were categorised according to the Charlson Comorbidity Index based on diagnoses five years before the emergency call. The Charlson Comorbidity Index is a combined score of previous medical conditions, with a score of 0 corresponding to no comorbidities, 1–2 to mild, 3–4 moderate and 5 or above as severe [15]. ICD-10 contains codes for diseases, presented in 22 chapters based on the underlying causes, such as diseases in the organ system (for example cardiovascular diseases), infections, cancer or external causes of injury. The ICD-10 chapters, R (‘symptoms, signs and abnormal clinical and laboratory findings, not elsewhere classified)’ and Z (*‘*factors influencing health status and contact with health services”) were combined and designated “non-specific diagnoses”. ICD-10 groups the diseases or conditions into chapters with associated main group and subgroups [16]. The main variables included were chapters and discharge diagnoses according to ICD-10 for patients assigned the Danish Index criterion ‘unclear problem’ and brought to the hospital with urgency levels A, B or C.

The prehospital data were linked to the hospital diagnoses, defined as hospital contact less than 5 h from the emergency call. Time of death is registered by date without time of day in the Danish Civil Registration System. Thus, we defined 1-day mortality as death within the same day as the emergency call or the following day. This approach was chosen to avoid underestimating the short-time mortality. We defined 30-day mortality to include all patients who died within 30 days, including 1-day mortality patients. Potential confounders were sex, age and comorbidities.

The diagnoses contributing most to the burden of disease were presented as the number of deaths and the proportion of the cumulative total number of deaths within 1 and 1–30 days.

The primary outcome was 1–30-mortality for the five ICD-10 chapters contributing most to the total number of deaths. In all results concerning mortality, we excluded 12 patients with diagnoses within the non-specific chapters e.g. ‘Other ill-defined and unspecified causes of mortality DR99’ i.e. the patients were not alive when they received these diagnoses.

### Data sources

Logistic data on the ambulance run and the Danish index were retrieved from the Logis CAD system (*Logis Solution A/S, Nærum, Denmark*).

Data on patient demographics, contact with the hospital, ICD-10 chapter and discharge diagnoses and comorbidities were obtained from the regional Patient Administrative System.

### Statistics

Descriptive statistics were used in terms of numbers and percentages for the distribution of ICD-10 chapters and discharge diagnoses along with age, gender, comorbidities and 1- and 30- day mortality.

Poisson regression with robust variance estimation was used to estimate proportions of mortality in percentages with 95% confidence intervals (CI), crude and adjusted for age, sex and comorbidities for the five chapters contributing most to the overall number of deaths.

All data were anonymised before analysis. Stata/MP 15.1 *(StataCorp LLC, Texas, United States of America)* was used for all statistical analyses.

### Ethics

This study was registered in the North Demark Region (ID 2020-038). The Danish Patient Safety Authority approved the disclosure of patient medical records (31-1521-299). According to Danish legislation, registry-based studies that do not involve biological material do not require approval from the National Committee on Health Research Ethics.

## Results

This study identified 95,237 emergency call patients from January 1st, 2016 to December 31st, 2018. A total of 4.3% (n = 4,132) patients were excluded due to missing civil registration number, 14.9% (n = 14,220) due to no hospital contact, 73.4% (69,950) patients due to other Danish Index criteria than ‘unclear problem’, and 9 (< 0.1%) patients with urgency level other than A, B, and C.

**Fig. 1:**
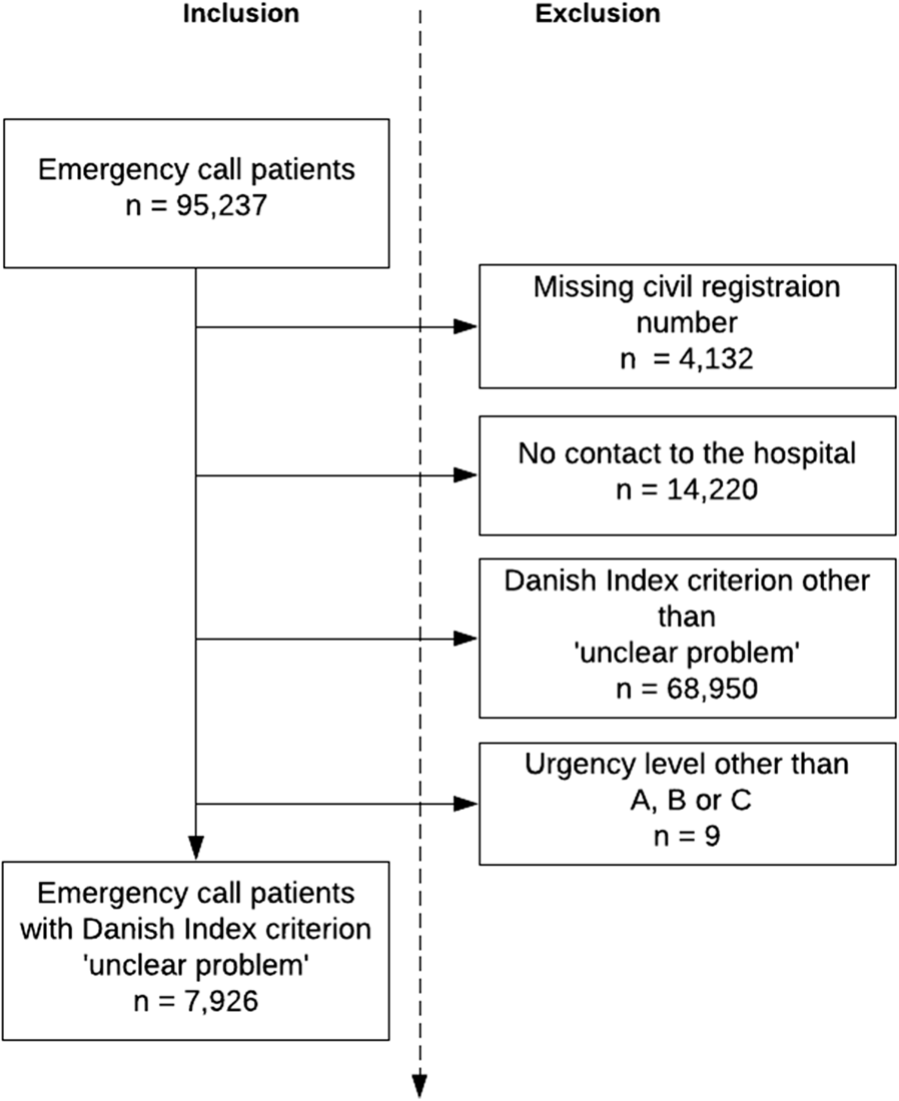
Data flowchart

A total of 7926 (8.3%) patients with Danish Index criteria ‘unclear problem’ were included in the study, the data flowchart is illustrated in Fig. 1.

**Fig. 2:**
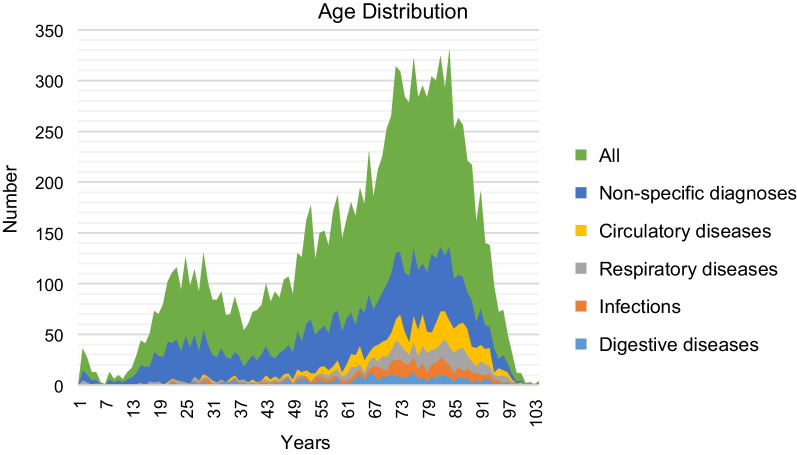
Age distribution for all emergency call patients with Danish Index criterion ‘unclear problem’ and the five ICD-10 chapters that contributed most to the number of deaths

The mean age was 61.5 years and 46,4% were females. Patients covered all age groups and the numbers increased with age with two distinct peaks among patients in their 20 s and patients in their 70 s and 80 s, The age distribution for all patients with ‘unclear problem’ is shown in Fig. 2, along with ICD-10 chapters that contribute most to the number of death.

The majority, 57.4% (n = 4,558) of the patients had no comorbidities, 29.4% (n = 2,329) patients had 1–2 comorbidities and 1,048 patients (13.2%) had 3 or more comorbidities.

Diagnoses included all ICD-10 chapters, except chapter 17 ‘certain conditions originating in the perinatal period’. The most frequent were ‘non-specific diagnoses’ (40.4%) ‘circulatory diseases’ (9.6%) ‘injuries and poisonings’ (9.4%), ‘respiratory diseases’ (6.9%), ‘mental disorders (6.2%) and ‘infections’ (5.1%), ‘digestive diseases’ (4.6%), ‘endocrine diseases’ (4.5%), ‘genitourinary diseases’ (3.8%), ‘musculoskeletal diseases’ (3.4%), ‘neurological diseases’ (3.3%), ‘neurological diseases’ (3.3%) and ‘ear diseases’, ‘neoplasms’, ‘blood diseases’, ‘pregnancy’, ‘skin diseases’, ‘eye diseases’ and ‘congenital diseases’ (< 1%).

A total of 2.3% of patients (n = 190) died on the same day as the emergency call or the following day and 7.1% (n = 554) died within 30 days. The ICD-10 chapters that contributed most to the total burden of deaths (n = 554) within 30 days were ‘circulatory diseases’ (n = 148, 26%) followed by ‘non-specific diagnoses’ (n = 88, 18%) ‘respiratory diseases’ (n = 85, 15.9%), ‘infections’ (n = 54, 10%) and ‘digestive disease’ (n = 39, 7%). These five diagnoses accounted for 75% of all deaths within 30 days. The ICD-10 chapters are presented in Additional file 1: Table S1 along with age, gender, comorbidities and 1- and 30-day mortality. 'Circulatory diseases’ showed the highest mortality in both numbers of all patients and percentages within the specific chapter.

Patients within the ICD-10 chapter ‘non-specific diagnoses’ had the lowest mean age and the lowest proportions of comorbidities among the five ICD-10 main chapters that contributed most to the number of deaths. The chapters with the highest number of comorbidities were ‘respiratory diseases’ and ‘infections’. An additional table shows this in more detail (see Additional file 1).

Table 1 shows crude and adjusted estimates for 1- and 30-day mortality in percentages for the five ICD-10 chapters contributing most to the number of deaths. Separate adjustments for age, sex and comorbidities showed great variations in affecting the risk of mortality. Overall high age had the greatest effect on the risk of mortality followed by comorbidities and sex (male), respectively. Age was also the only factor that demonstrated a significant effect on mortality as there was no overlap of CIs. Sex showed the least effect on mortality with similar estimates with overlapping CIs. Adjusted for all three confounders, the greatest reduction in mortality was in ‘circulatory diseases’ for 30-day mortality which decreased from 19.4% (95%CI 16.8–22.1) to 1 0.2% (95% CI 0.6–1.9) and the smallest reduction was in ‘non-specific diagnoses’ for 30-day mortality decreased from 3.1% (95% CI 2.6–3.8) to 0.1% (95% CI 0.2–0.5).

**Table 1:** Mortality estimates crude and adjusted for age, sex and comorbidities mortality estimates for 1-day and 30-day mortality in percentages (%) with 95% CI

ICD-10 chapter	Crude %(95%CI)	Adjusted			
		Age %(95%CI)	Sex %(95%CI)	Comorbidities %(95%CI)	Age, sex and comorbidities %(95%CI)
*1-day mortality rate estimates*					
Circulatory diseases	8.7 (6.9 -10.9)	0.5 (0.2–1.3)	7.9 (5.9–10.7)	6.4 (4.7–8.7)	0.4 (0.2–1.3)
Infections	5.4 (3.6–8.2)	0.3 (0.1–0.8)	5.0 (3.1–8.1)	3.7 (2.4–5.8)	0.4 (0.1–1.0)
Respiratory diseases	4.0 (2.7–6.0)	0.2 (0.1–0.6)	3.7 (2.3–5.8)	2.8 (1.7–4.5)	0.3 (0.0–0.7)
Digestive diseases	3.0 (1.7–5.4)	0.2 (0.1–0.5)	2.8 (1.5–5.2)	2.2 (1.2–4.2)	0.2 (0.0–0.7)
Non-specific diagnoses	0.9 (0.6–1.4)	0.1 (0.1–0.1)	1.3 (0.9–1.8)	1.1 (0.8–1.5)	0.1 (0.0–0.2)
*30-day mortality rate estimates*					
Circulatory diseases	19.4 (16.8–22.1)	1.1 (0.6–2.0)	19.1 (15.9–22.9)	12.5 (10.3–15.1)	1.2 (0.6–1.9)
Infections	13.2 (10.4–17.1)	0.9 (0.5–1.5)	13.1 (10.0–17.3)	7.7 (5.8–10.3)	0.7 (0.4–1.3)
Respiratory diseases	15.5 (12.2–18.8)	1.0 (0.5–1.7)	15.2 (12.1–19.2)	9.0 (7.1–11.5)	0.8 (0.4–1.5)
Digestive diseases	10.7 (8.0–14.5)	0.8 (0.4–1.4)	10.6 (7.8–14.4)	6.9 (5.1–9.5)	0.7 (0.4–1.3)
Non-specific diagnoses	3.1 (2.6–3.8)	0.3 (0.2–0.5)	3.1 (2.5–3.8)	2.3 (1.9–2.8)	0.3 (0.2–0.5)

## Discussion

The aim of this study was to investigate which diagnoses contributed most to the total burden of diseases in terms of numbers of deaths together with 1- and 30-day mortality. The diagnoses with the highest numbers of death were ‘circulatory diseases’ (n = 148, 26%) followed by ‘non-specific diagnoses’ (n = 88, 18%) ‘respiratory diseases’ (n = 85, 15.9%), ‘infections’ (n = 54, 10%) and ‘digestive disease’ (n = 39, 7%). The overall mortality was 2.3% (1 day) and 7.1% (30-days). The mortality estimates showed that circulatory diseases had the highest risk of mortality, followed by infection, respiratory diseases, digestive diseases, and lastly non-specific diagnoses. Even though patients with non-specific diagnoses had low 1- and 30 days mortality rates, they contributed considerably to the number of deaths as the second highest of all ICD-10 chapters. All mortality estimates were significantly reduced when adjusted for age.

A strength of this study was the thorough follow-up, which was possible due to the use of the Danish registries, which reduced the risk of bias. The free access to healthcare including EMS in Denmark ensured equal availability for all citizens, which reduces selection bias due to financial differences between the included patients. In the present study, it was not possible to clarify the causes of the assessment of the criterion ‘unclear problem’. There may also be other factors underlying ‘unclear problem’, such as language barriers or lack of information from the caller, poor phone connection, or patients presenting with symptoms not classified elsewhere. Which should be investigated in future studies.

The broad diagnostic spectrum of underlying diseases corresponds well to what has previously been described in an observational ED study from Switzerland [5], the BANC (Basel Nonspecific Complaints) study. That study showed that underlying diagnoses were classified across 18 of 22 ICD-10 chapters for patients presenting in the ED with non-specific symptoms. Furthermore, the BANC study found similar 30-day mortality (6.4%) as our study (7,1%). However, patients in the BANC study were included after history-taking and a focused clinical examination in the ED which contrasts with the telephone assessment in our study. This likely explains the difference in the proportion of patients with ‘non-specific diagnoses’ which was 9% in the Swiss study compared with 40% in our study.

The higher likelihood to receive a non-specific diagnosis after an emergency presentation with an ‘unclear problem’ has previously been demonstrated in a study from the Danish Capital Region. That study found similar results as 46% of patients with ‘unclear problem’ from prehospital dispatchers were discharged with non-specific diagnoses [17]. The large proportion of patients discharged with non-specific diagnoses after examination, blood tests, imaging and other diagnostics at the hospital indicates that this was indeed an ‘unclear problem’ as assessed by the emergency call. However, we do not have information about the in-hospital diagnostic process, and whether there may be room for improvement here.

Patients with ‘non-specific diagnoses’ had a lower mean age and lower number of comorbidities, compared to the chapters ‘circulatory diseases’, ‘infections’, ‘respiratory diseases’ and ‘digestive diseases. The non-specific diagnoses had the second-highest number of deaths, despite the 30-day mortality percentage being relatively low (3%). These results show that within the large group of patients with ‘non-specific diagnoses’ there are patients who are seriously ill and future studies should focus on how to identify these patients.The overall 30-day mortality for patients with ‘unclear problem’ was 7.1% which corresponds to the 6.8% 30-day mortality for all emergency call patients, which we previously demonstrated. However, the overall 1- day mortality for patients with ‘unclear problem’ (2.3%) was lower than for all emergency call patients ( 3.9%) [18]. This study demonstrated that ‘non-specific diagnoses’ had the second highest number of deaths. This indicates that within this group, there are patients who, despite a severe condition, have not received a specific diagnosis that could explain the cause of death. There is a need for further analysis of the in-hospital diagnoses to understand the underlying conditions*,* e.g. by performing a medical record audit.Our study showed that the high mortality among patients with ‘unclear problem’ was predominately associated with higher age. There was no clear association between mortality and sex. Though, there was a small tendency for men to have an increased risk of mortality compared to women for all diagnoses except the non-specific diagnoses. The mean age of the patients with ‘unclear problem’ in this study was 61.5 years compared to 55.3 years previous demonstrated for all patients with contact to Emergency department [19]. The older age of patients with ‘unclear problem’ corresponds to what has previously been shown for patients presenting to the ED with non-specific complaints [5, 20, 21]. However, our study showed that patients with an ‘unclear problem’ at the emergency call and who ended up with a non-specific diagnosis had a lower mean age than those with ‘unclear problem’ and specific diagnoses. Furthermore, the mortality estimates decreased significantly when adjusted for age for patients with non-specific diagnoses. Further research is needed to better understand the different patient groups, within the group of patients with ‘unclear problem’ and nonspecific diagnoses, as it seems to be a mixed population comprising both high and low-risk patients. This population seems to represent a group of patients in whom a clear treatable diagnosis has not been made (e.g. due to delay), or even a failure to make a clear diagnosis that might not be trated in the same way as a patient with a circulatory or respiratory diagnosis. There are several similarities between the non-specific complaints seen in the EDs and ‘unclear problem’ presented when calling EMS. First, the patients are older, have more comorbidity and have an increased risk of death. Second, the categories of non-specific and unclear problems are chosen in situations when a differential diagnosis or criterion based on the available information is not possible. Additionally, we do not know whether the patients had specific symptoms when assessed by the ambulance personnel. A previous Danish register-based study with data from 2011 to 2013 from the Capital Region of Denmark found that age, ethnicity, comorbidity, time of day, time of the week, employment, and education level were significant predictors for the emergency call being categorised as ‘unclear problem’ [17].

## Conclusion

This study found that patients brought to the hospital after calling 112 with an ‘unclear problem’ were discharged with a large variety of diagnoses. Almost half of the patients were discharged with a ‘non-specific diagnosis’ which might seem trivial but should be explored more as these contributed the second-highest to the total number of deaths after 30 days only exceeded by ‘circulatory diseases’. Further research is needed to explore the group of patients with ‘unclear problem’ and nonspecific diagnoses, as it seems to be a mixed population comprising both high and low-risk patients.

## Supplementary Information

**Additional file 1**. **Table S1:** The most frequent ICD-10 chapters.

## Data Availability

As the study include sensitive patient information, restrictions apply to the availability of data that is not publicly available. However, researchers interested in the data can seek approval from the Danish Patient Safety Authority. Having obtained approval, researchers can request data from the North Denmark Region, Centre of Prehospital and Emergency Research.

## Abbreviations

ICD-10: International statistical classification of diseases and related health problems, 10th edition
ED: Emergency department
EMS: Emergency medical services
Danish Index: Danish index for emergency care

## References

[1] Nemec M, Koller MT, Nickel CH, Maile S, Winterhalder C, Karrer C (2010). Patients presenting to the emergency department with non-specific complaints: the basel non-specific complaints (BANC) study. Acad Emerg Med.

[2] Peng A, Rohacek M, Ackermann S, Ilsemann-Karakoumis J, Ghanim L, Messmer AS (2015). The proportion of correct diagnoses is low in emergency patients with nonspecific complaints presenting to the emergency department. Swiss Med Wkly.

[3] Djärv T, Castrén M, Mårtenson L, Kurland L (2015). Decreased general condition in the emergency department. Eur J Emerg Med..

[4] Ivic R, Kurland L, Vicente V, Castrén M, Bohm K (2020). Serious conditions among patients with non-specific chief complaints in the pre-hospital setting: a retrospective cohort study. Scand J Trauma Resusc Emerg Med..

[5] Karakoumis J, Nickel CH, Kirsch M, Rohacek M, Geigy N, Muller B (2015). Emergency presentations with nonspecific complaints-the burden of morbidity and the spectrum of underlying disease: nonspecific complaints and underlying disease. Med.

[6] Ibsen S, Lindskou TA, Nickel CH, Kløjgård T, Christensen EF, Søvsø MB (2021). Which symptoms pose the highest risk in patients calling for an ambulance? A population-based cohort study from Denmark. Scand J Trauma Resusc Emerg Med.

[7] Rutschmann OT, Chevalley T, Zumwald C, Luthy C, Vermeulen B, Sarasin FP (2005). Pitfalls in the emergency department triage of frail elderly patients without specific complaints. Swiss Med Wkly.

[8] Pittet V, Burnand B, Yersin B, Carron P-N (2014). Trends of pre-hospital emergency medical services activity over 10 years: a population-based registry analysis. BMC Health Serv Res.

[9] Danish Regions. Dansk Indeks for Akuthjælp [Danish Index for Emergency Care]. Ver. 1.8. Laerdals Fond for Akuttmedisin; 2017 [cited 2019 Nov 11]. Available from: https://pri.rn.dk/Assets/30522/Dansk-Indeks-1-8-Region-Nordjylland.pdf.

[10] Andersen MS, Johnsen SP, Sørensen JN, Jepsen SB, Hansen JB, Christensen EF (2013). Implementing a nationwide criteria-based emergency medical dispatch system: a register-based follow-up study. Scand J Trauma Resusc Emerg Med.

[11] Otten S, Rehbock C, Krafft T, Haugaard MV, Pilot E, Blomberg SN (2022). The “unclear problem” category: an analysis of its patient and dispatch characteristics and its trend over time. BMC Emerg Med..

[12] Vandenbroucke JP, Von Elm E, Altman DG, Gøtzsche PC, Mulrow CD, Pocock SJ (2007). Strengthening the reporting of observational studies in epidemiology (STROBE): explanation and elaboration. PLoS Med.

[13] Lindskou TA, Mikkelsen S, Christensen EF, Hansen PA, Jørgensen G, Hendriksen OM (2019). The Danish prehospital emergency healthcare system and research possibilities. Scand J Trauma Resusc Emerg Med..

[14] Statistics Denmark. FOLK1A: Folketal den 1. i kvartalet efter område, køn, alder og civilstand [FOLK1A: Population the 1. of the quarter, by area, sex, age and marital status]. 2020. Available from: https://www.statistikbanken.dk/FOLK1A.

[15] Charlson ME, Pompei P, Ales KL, MacKenzie CR (1987). A new method of classifying prognostic comorbidity in longitudinal studies: development and validation. J Chronic Dis.

[16] World Health Organization. International Statistical Classification of Diseases and Related Health Problems 10th Revision. 2019 [cited 2019 Apr 11]. Available from: https://icd.who.int/browse10/2016/en.

[17] Møller TP, Kjærulff TM, Viereck S, Østergaard D, Folke F, Ersbøll AK (2017). The difficult medical emergency call: A register-based study of predictors and outcomes. Scand J Trauma Resusc Emerg Med..

[18] Ibsen S, Lindskou TA, Nickel CH, Kløjgård T, Christensen EF, Søvsø MB (2021). Which symptoms pose the highest risk in patients calling for an ambulance? A population-based cohort study from Denmark. Scand J Trauma Resusc Emerg Med..

[19] Søvsø MB, Hermansen SB, Færk E, Lindskou TA, Ludwig M, Møller JM (2018). Diagnosis and mortality of emergency department patients in the North Denmark region. BMC Health Serv Res.

[20] Wachelder JJH, Stassen PM, Hubens LPAM, Brouns SHA, Lambooij SLE, Dieleman JP (2017). Elderly emergency patients presenting with non-specific complaints: Characteristics and outcomes. PLoS One.

[21] Lucke JA, Mooijaart SP, Conroy S, Blomaard LC, De Groot B, Nickel CH (2021). Mortality risk for different presenting complaints amongst older patients assessed with the Manchester triage system. Eur Geriatr Med.

