# Supplementary material for: Diagnoses and mortality among prehospital emergency patients calling 112 with unclear problems: a population-based cohort study from Denmark
Source: Scand J Trauma Resusc Emerg Med. 2022 Dec 12;30:70. doi: 10.1186/s13049-022-01052-y (PMC9743502; doi:10.1186/s13049-022-01052-y)
Supplement: Supplementary file 1 — Additional file 1. Table S1: The most frequent ICD-10 chapters. [file 13049_2022_1052_MOESM1_ESM.docx]

Table 1: The most frequent ICD-10 chapters

| ICD-10 chapter | Frequency  n (%) | Age  mean (95%CI) | Female  n(%) | 1-2 comorbidities  n(%) | 3 or more comorbidities  n(%) | 1-day mortality  n(%) | 30-day mortality  n(%) |
| --- | --- | --- | --- | --- | --- | --- | --- |
| Circulatory diseases | 763(9.6) | 73.4(72.41-74.32) | 320(41.9) | 254(11.0) | 129(12.4) | 66(39,1) | 148(26.7) |
| Non-specific diagnoses | 3,202(40.5) | 56.7(56.08-57.69) | 1,600(50.0) | 770(33.2) | 305(29.2) | 32(18.9) | 88(15.9) |
| Respiratory diseases | 549(6.9) | 69.6(67.9-71.29) | 234(42.6) | 215(9.3) | 127(12.2) | 22(13.1) | 85(15.3) |
| Infections | 405(5.1) | 69.8(68.2-71.5) | 168(41.5) | 163(7.0) | 94(9.0) | 22(13.1) | 54(9.9) |
| Digestive diseases | 363(4.6) | 64.4(62.41-66.38) | 179(49.3) | 104(4.5) | 66(6.3) | 11(6.5) | 39(7.2) |
| Remaining chapters | 2,632(33.3) | 64.4(63,5-65,4) | 8,37(47,2) | 598(25.8) | 287(27.5) | 25(14.8) | 133(24.4) |
| All Chapters | 7926(100) | 61.5(61.0-61.9) | 3,673(46,4) | 2,320(100) | 1,044(100) | 169(100) | 554(100) |

Table 1: The most frequent ICD-10 chapters (>5%); frequency, age, gender, comorbidities, 1- and 30-day mortality, sorted by the cumulative number of deaths
